# ‘I still don’t know diddly’: a longitudinal qualitative study of patients’ knowledge and distress while undergoing evaluation of incidental pulmonary nodules

**DOI:** 10.1038/npjpcrm.2015.28

**Published:** 2015-04-16

**Authors:** Donald R Sullivan, Sara E Golden, Linda Ganzini, Lissi Hansen, Christopher G Slatore

**Affiliations:** 1 Health Services Research and Development, Veterans Affairs Portland Health Care System, Portland, OR, USA; 2 Division of Pulmonary and Critical Care Medicine, Department of Medicine, Oregon Health and Science University, Portland, OR, USA; 3 Division of Geriatric Psychiatry, Department of Psychiatry, Oregon Health and Science University, Portland, OR, USA; 4 School of Nursing, Oregon Health and Science University, Portland, OR, USA; 5 Section of Pulmonary and Critical Care Medicine, Veterans Affairs Portland Health Care System, Portland, OR, USA

## Abstract

**Background:**

Hundreds of thousands of incidental pulmonary nodules are detected annually in the United States, and this number will increase with the implementation of lung cancer screening. The lengthy period for active pulmonary nodule surveillance, often several years, is unique among cancer regimens. The psychosocial impact of longitudinal incidental nodule follow-up, however, has not been described.

**Aims:**

We sought to evaluate the psychosocial impact of longitudinal follow-up of incidental nodule detection on patients.

**Methods:**

Veterans who participated in our previous study had yearly follow-up qualitative interviews coinciding with repeat chest imaging. We used conventional content analysis to explore their knowledge of nodules and the follow-up plan, and their distress.

**Results:**

Seventeen and six veterans completed the year one and year two interviews, respectively. Over time, most patients continued to have inadequate knowledge of pulmonary nodules and the nodule follow-up plan. They desired and appreciated more information directly from their primary care provider, particularly about their lung cancer risk. Distress diminished over time for most patients, but it increased around the time of follow-up imaging for some, and a small number reported severe distress.

**Conclusions:**

In settings in which pulmonary nodules are commonly detected, including lung cancer screening programmes, resources to optimise patient-centred communication strategies that improve patients’ knowledge and reduce distress should be developed.

## Introduction

In the United States, hundreds of thousands of incidental pulmonary nodules are detected annually during chest imaging.^1–3^ This number is expected to increase substantially because lung cancer screening is now recommended by the United States Preventative Services Task Force^4^ for patients with elevated risk, defined as adults aged 55–80 years who have a 30-pack-year smoking history and currently smoke or have quit within the past 15 years, on the basis of the results of the National Lung Screening Trial.^5^ Once detected, experts recommend that patients with nodules undergo serial follow-up imaging, often for 2 years.^6,7^ Researchers are just beginning to explore potential psychological harms of nodule detection^8^ and how these harms may be mitigated.

Patients experience significant distress after incidental nodule detection.^9–11^ A systematic review of lung cancer screening trials, mostly from Europe, found that false positive screening results were often associated with short-term increases in distress, which returned to baseline levels over time.^8^ Little is known about how patients’ emotional responses, knowledge of an incidental pulmonary nodule and the follow-up plan change over time, as well as the impact of distress. In other settings not related to pulmonary nodule detection, distress impedes smoking cessation and contributes to re-initiation.^12,13^ Among patients undergoing breast and colorectal cancer screening, distress interferes with adherence to subsequent medical care and screening recommendations.^14–18^

Provider communication strategies that help mitigate patients’ distress and improve comprehension are important, as recommended by a bioethics commission on incidental findings.^19^ Learning how these strategies satisfy patients’ expectations, preferences and desires is important, as they are essential components of quality care.^20,21^ We previously reported the results of a qualitative analysis of veterans’ knowledge and distress of initial pulmonary nodule detection.^22^ The present study extends that work through follow-up interviews over 2 years to explore patients’ understanding of the pulmonary nodule and its evaluation process, their emotional reactions and their views about provider communication regarding nodule detection. Exploring patients’ longitudinal experiences is essential given the potential impact on adherence to medical care, smoking cessation and their well-being over time. In addition, the unique length of follow-up recommended for pulmonary nodules, compared with other cancer screening programmes, makes longitudinal results particularly salient.

## Materials and methods

A follow-up qualitative study was conducted at the VA Portland Health Care System, which is an academically affiliated hospital with outlying clinics, among 17 patients with an incidentally detected (not from screening) pulmonary nodule. At the VA Portland Health Care System, radiology images with pulmonary nodules are electronically flagged.^1^ Among patients with small nodules, primary care providers (PCPs) are usually responsible for notification and determination of the timing of subsequent evaluations without guidance from pulmonologists. PCPs included were physicians, nurse practitioners and physician assistants. Asymptomatic patients with a plan to obtain non-urgent follow-up imaging were eligible. We excluded patients who scored <17/30 on the Saint Louis University Mental Status Examination,^23^ who resided in skilled nursing care facilities and who were diagnosed with psychotic or cognitive disorders, a terminal illness or severe hearing impairment. After approval from respective patients’ PCPs and mental health clinician (if relevant), we contacted the patient by mail to invite participation. The Internal Review Board of the VA Portland Health Care System approved this study and all the patients provided written informed consent.

Participants were interviewed after first and second annual follow-up chest CT scans for a maximum of two follow-up interviews or ~2 years. Interviews stopped when nodule follow-up imaging was no longer recommended. As a result, 17 of the initial 19 participants were interviewed after their first annual follow-up CT scan, and 6 of these were interviewed after their second annual follow-up scan. After the baseline interview, one patient died from an unrelated medical problem and one declined further participation. None of the participants missed imaging appointments or were lost to follow-up. The interview guide (Supplementary File) was organised around the core domains of the patient-centred communication (PCC) model, which emphasises the importance of the relationship between communication and health. Key domains of effective PCC include the following: Information Exchange, Patient as Person, Sharing Power and Responsibility, Therapeutic Alliance and Provider as Person.^24^ Participants were interviewed by an experienced qualitative interviewer (CGS, a pulmonologist), and none of the participants had a previous relationship with the interviewer. Interviews were digitally recorded. We recorded self-reported demographic and smoking characteristics; nodule characteristics were based on imaging reports and medical records review, not independent review of the images. Participants were assigned study letters, and numbers were used to indicate from which visit quotes were obtained: V2=visit two, V3=visit three.

### Analysis

We used conventional content analysis, which allows comprehensive description of a patient’s experience in everyday language with little dependence on interpretation or theorisation.^25,26^ Data were reviewed and analysed in two separate but comparable phases. Phase 1 included the baseline patient interviews only^22^ and phase 2 included all follow-up interviews. The code structure was developed inductively^27^ using the PCC model as an organising framework.^24^ The phase 2 analysis included a comparison of codes between baseline and follow-up interviews.^26^ Follow-up interviews were reviewed by CGS, DRS and SEG, who as a group reviewed two completed patient transcripts to assess congruence with the codebook developed from the phase 1 analysis. The codes began with, and remained close to, the questions of the initial interview guide, with adjustments for a longitudinal perspective, that explored the Veterans’ experiences receiving information, concerns and emotional responses, desire for more information and ideas for improvement. Additional codes were added for issues and concerns that arose in reviewing these transcripts, leading to a more robust codebook—e.g., ‘more, less or the same’ under the theme of longitudinal changes in level of distress. After the codebook was adjusted, DRS and SEG independently reviewed and coded the original two transcripts and an additional two transcripts. CGS, DRS and SEG then reviewed the same four transcripts to discuss and assess discrepancies. We achieved more than our predetermined 80% level of agreement demonstrating trustworthiness of the analyses. A consensus process was used to resolve disagreements, adjudicated by the principal investigator (CGS). Saturation of most qualitative themes was achieved. The remaining interviews were independently coded by DRS and SEG using the established codebook. ATLAS.ti (Berlin, Germany) was used to organise the data.

## Results

Participating veterans were mostly older, white men with smoking histories (Table 1). None of them were diagnosed with lung cancer at the time of final analysis. Primary care clinicians were responsible for the care related to the nodule for all the participants. The average time from nodule diagnosis to baseline, second and third interview was 154, 438 and 648 days, respectively, which did not seem to influence the participants’ responses. Participants’ responses were organised into five major themes: Patient Knowledge, Emotional Response/Distress, Communication with the Primary Care Provider, Suggestions for Improvement, and Impact of Research Participation.

### Patient knowledge

All the participants reported receiving either a telephone call or a letter relaying the results of their follow-up CT scans from their PCPs. These letters often contained ‘cut/paste’ phrases from the radiology report notifying participants of imaging results. Sometimes this letter contained information regarding the subsequent follow-up plan at the discretion of patients’ PCPs. In most cases, participants reported that this letter was confusing, did not contain enough information and did not allow an opportunity to ask questions. Similarly, most participants who received telephone calls reported that they had inadequate information about the nodule.

Over time, the participants rarely elicited more information about the nodule from their PCP because they trusted their PCP and/or took cues from them that influenced their information-seeking behaviours. In many instances, PCPs were solely responsible for ensuring adequate monitoring of the nodule without patient involvement in decision-making regarding follow-up imaging. We found that when PCPs did not communicate the follow-up plan, participants assumed that this meant no follow-up was planned. Some participants did not seek additional information because they felt adequately informed by their PCP. Other reasons participants expressed for not seeking more information from their PCPs were fears of knowing in relation to lung cancer and the prioritisation of other medical problems. Similar to findings from baseline interviews, several participants considered the nodule a low priority compared with other active or symptomatic medical diseases.

Despite several opportunities to increase participant knowledge from the initial nodule detection to follow-up notifications from PCPs, most participants expressed a persistent lack of knowledge regarding what a nodule is and the follow-up plan for future imaging. The main themes expressed by most participants at follow-up was confusion about what a nodule is, what symptoms the nodule could cause, and what procedures might occur after being told about the existence of the nodule (e.g., biopsy). A few participants did find out more information about their nodule, primarily through contact with their PCP or through their involvement in the research study (Table 2). A small number of participants obtained additional information from the library, or family members with knowledge or past experiences with pulmonary nodules. Some participants reported that online information was unhelpful because it was difficult to understand or not relevant to their situation.

### Emotional response/distress

During follow-up interviews, many participants reported mild persistent distress regarding their nodule, particularly around the time of follow-up imaging. Participants’ lung cancer concerns seemed to remain the primary contributor to this persistent distress during follow-up. When discussing distress related to the nodule, Veteran A-V3 stated that ‘I’m 84, I’m going to die of something, but I’d really not [like to] die of lung cancer.’ Even though the participants did have a lack of understanding of pulmonary nodules, most associated them with lung cancer as Veteran P-V2 stated ‘…I still don’t really understand what they [nodules] are. But I assume they’re associated with lung cancer. And that’s all I know.’ The association between the nodule and participants’ lung cancer concerns were mainly driven by current or previous smoking history as Veteran G-V2 stated, ‘I’m a good candidate for cancer, I smoked for years.’

Overall, most participants reported that their distress decreased since the initial nodule detection (Table 3). Among participants who reported moderate/severe distress during the initial interview, most of them also reported that their distress had decreased during follow-up. In those who reported persistent distress, the length of time waiting for follow-up CT scans and results was a common cause of concern. Over time, participant distress was mitigated owing to several factors, including a lack of symptoms attributed to the nodule, favourable follow-up CT results and patients’ perception that their provider was not worried about the nodule. Many participants reported taking cues, both verbal and nonverbal, from their PCPs, which decreased their distress. If the PCPs ‘didn’t sound anxious or anything’ or were perceived as ‘really reassuring’ and ‘very positive’ when delivering results, participants were reassured and reported decreased distress.

Themes regarding the impact of distress on participants’ decisions regarding smoking cessation did not reach saturation, as few participants were current smokers. Veteran C-V2 felt if the nodule was lung cancer, then there was no reason to consider quitting ‘…if I was diagnosed with lung cancer and I’ve got 6 months to live, I’d probably still smoke.’ Veteran J-V2 used the nodule detection as a teachable moment to inspire previous unsuccessful cessation attempts, ‘Sure, emphysema isn’t all that good to have, but it’s not cancer. I’m making a concerted effort right now to quit [smoking].’

### Communication with the primary care provider

During follow-up, participants who had face-to-face or telephone discussions with their PCP regarding nodule changes appreciated and desired this mode of communication, as it afforded a back-and-forth exchange and opportunity to ask questions. These follow-up interactions were just as important to participants as their initial nodule notifications. Some participants also reported that the length of their relationship and previous positive experiences with their PCP helped augment effective nodule communication. Most participants found reassurance and trust were the key components of the communication they desired. Veteran P-V2 explained, ‘I trust my primary care doctor. If [my PCP] told me I had nodules and they are no big deal, then I believe [my PCP].’

There were several provider and participant-related barriers to effective communication identified during follow-up. Some participants felt their PCPs were too busy and were difficult to contact. New participant-related barriers at follow-up included the following: participants forgot to ask their PCP about the nodule during routine office appointments or reasoned the nodule was insignificant and not worth discussing because they were asymptomatic over time, ‘Well I’m curious of course, but it’s not causing me any immediate discomfort or anything like that so you don’t know it’s there,’ and ‘Oh well I don’t know. As long as it’s not interfering with my life or anything, who gives a shit?’ as Veteran N-V2 stated. During follow-up interviews, participants continued to take cues from their PCPs during nodule discussions at visits, reasoning that if PCPs did not bring it up, the nodule must not be significant.

### Patients’ suggestions for improvement

Participants’ dislike of letter notification for CT scan results extended from the initial nodule detection to follow-up notifications. Most participants indicated that letters were appropriate if they were followed up by direct communication with a provider. Suggestions for letter notification improvements included providing a rating or scale regarding the amount a subject should be concerned about the nodule. During follow-up, participants wanted more information about how a nodule is managed, and asked for information on signs and symptoms of a nodule that should prompt more urgent medical care. Participants desired more information on the risk of lung cancer (Table 4).

### Impact of research participation

We hypothesised that contacting potential participants via study letter might increase their distress about their nodule, and interviews might actually heighten these emotions. Some participants reported that this was the effect of the baseline study letter: ‘The worry really started coming, the actual worry started coming is when I got the letter from you guys [research]’ as Veteran Q-V2 stated. However, participation in the interviews over time seemed to decrease distress for these same patients: ‘By you [research] telling me that it’s unnecessary to do another CT scan and that basically it shouldn’t grow anymore, that’s encouraging’ as Veteran F-V3 stated, and ‘...cause you guys [research] told me that they hardly ever grow and stuff. So after meeting you that was a positive experience- you guys are alright’ as veteran D-V2 stated. Overall, study interviews seemed to decrease participants’ distress.

## Discussion

### Main findings

We previously reported that Veterans with incidental pulmonary nodule did not understand the term ‘nodule’ or the follow-up plan and most experienced distress, related to fears of malignancy, although they were unaware of their lung cancer risk. This study is the first to provide longitudinal qualitative information among patients with incidentally detected pulmonary nodules. Despite up to 2 years of follow-up, many patients had persistently inadequate knowledge of pulmonary nodules, the follow-up plan and their lung cancer risk. Providers’ communication strategies, mostly mail notification, may have contributed to patients’ passive role in information-seeking, which probably diminished their knowledge. Patients’ distress decreased over time from the initial detection of the nodule; however, it often increased around the time of follow-up imaging and was persistent in others. Patients overwhelmingly preferred direct, personal communication. Patients particularly valued reassurance, an accurate discussion of lung cancer risk, and the opportunity to ask questions about the nodule.

### Interpretation of findings in relation to previously published work

Patients’ have been previously shown to have emotional responses to incidental nodule detection.^9–11,22^ In addition, providers’ initial communication regarding nodule detection does affect patients’ perceptions and distress.^9^ Distress of this type may lead to poor adherence with further evaluations, as has been described in the context of screening for other cancers.^14–18^ Our patients had a poor understanding of the follow-up plan, which may predispose them to nonadherence with medical evaluations; however, their participation in our study biased any examination of adherence. Neither longitudinal changes in patients’ distress over time nor the impact of patients’ knowledge of an incidental nodule and the follow-up plan on their distress have been previously examined. The psychosocial impact of longitudinal incidental nodule follow-up deserves greater attention considering the potential consequences on patients’ well-being, such as distress, and adherence to subsequent medical care. The abundance of incidental pulmonary nodules detected annually in the United States^2,3^ demonstrates the significance of our findings.

Pulmonary nodules are small (<3 cm), rounded, well-circumscribed radiographic opacities surrounded by normal aerated lung.^28^ Nodule follow-up is based on lung cancer risk, which is most dependent on nodule size and patients’ smoking history.^6^ As most small nodules are not early lung cancer and the risk of spread is low over a short time interval, most guidelines recommend active surveillance with chest imaging instead of invasive procedures.^6,7^ On the basis of the lung cancer mortality benefit of the National Lung Screening Trial,^5^ multiple organisations recommend lung cancer screening to high-risk individuals. It is estimated that 8.6–10 million Americans would meet the National Lung Screening Trial criteria for lung cancer screening annually.^29,30^

### Implications for future research, policy and practice

Overwhelmingly, patients wanted more information about their pulmonary nodule and their risk of lung cancer. To enhance this process, it is important to ask patients what they expect at the outset of the encounter to help define roles and to prevent assumptions.^31^ Patients preferred direct to indirect communication in this and previous studies,^9^ and patients felt notification letters should serve as an adjunct and not as a replacement for direct communication. Communication about risks is difficult, as common terms such as probably, unlikely and rarely are not well defined or understood.^32^ Instead, portrayal of risk and satisfaction with decisions may be enhanced using decisional aids, data depicted in pictures, or summary tables.^32,33^ Wide variations exist in provider communication strategies for delivering bad news,^34–36^ but all participants made a connection between the nodule and lung cancer despite a lack of knowledge. Therefore, the inclusion of lung cancer risks should be a key component of nodule discussions. Systems-based resources should be developed to support providers to engage in PCC. These resources may include outlines for providers highlighting key strategies for enhanced communication, as well as patient educational materials. More research is needed particularly among health-care systems with large lung cancer screening programmes to help develop evidence-based resources for providers and patients. Using patients’ suggestions, preferences and addressing their concerns may serve as an organising framework for PCC strategies during the evaluation process (Table 5).

### Strengths and limitations of this study

There are study limitations. This study was conducted among mostly male, elderly veterans. We enrolled patients only after permission from their PCP, perhaps creating a selection bias for patients with more knowledge and better communication. The time between the interview and imaging study was variable, and thus recall bias may influence findings. Several participants indicated that their distress decreased as a result of study interviews, likely causing us to underestimate distress in clinical settings. Patients’ perceived quality of communication may be affected by their lung cancer concerns. Patients with probable lung cancer often report lower communication scores with providers and are more likely to feel they have not had enough time or opportunity to voice distress and ask questions.^37^ Therefore, patients’ lung cancer concerns, which were prevalent, may have affected perceived communication. Owing to sample size constraints, themes regarding the impact of distress on participants’ decisions regarding smoking cessation did not reach saturation, which may reduce the quality of this evidence.

### Conclusions

Developing systematic resources to improve patients’ knowledge, reduce their distress and refine PCPs’ communication strategies in order to enhance patients’ satisfaction, adherence and outcomes should be essential components of lung cancer screening programmes and other settings where pulmonary nodule detection is common.

## Figures and Tables

**Table 1:** Follow-up cohort characteristics

*Characteristic*	*Statistics*
	N *(%) or mean (±s.d.)*
Age (years)	64 (±11)
	
*Gender*	
Male	16 (94)
	
*Race/ethnicity*	
White	14 (82)
	
*Smoking status*	
Current smoker	4 (24)
Former smoker	9 (53)
Never smoker	4 (24)
	
*Education*	
High school or less	5 (29)
Largest nodule size (in diameter)	5 mm (±3 mm)
	
*Type of primary care provider* a	
Physician	14 (82)
Nurse practitioner	2 (12)
Physician assistant	1 (6)
	
*CT scans completed, (% of initial cohort)*	
Baseline	19 (100)
Second^b^	17 (89)
Third^c^	6 (32)
	
*Length of time (days) from nodule detection to:*	
Second interview	438 (±63)
Third interview	648 (±79)
	
*Length of time (days) from CT scan to:*	
Second interview	78 (±42)
Third interview	56 (±20)

Percents are of non-missing information and may not add up to 100% owing to rounding.

Abbreviation: CT, computed tomography.

a Each participant had a unique primary care provider.

b One participant died and one participant withdrew.

c Eleven participants from the second interview did not undergo further imaging.

**Table 2:** Effectiveness of increasing patient knowledge

*Continued lack of knowledge*	
Veteran N-V2	‘Right now I don’t know diddly about what it is or what may have caused it or, you know. I just don’t know.’
Veteran B-V2	‘What possible future health risks they may cause? Like I said earlier, are they gonna mutate into something like a tumor or are they gonna be just a lump? Like a cyst? That’s what I’d really like to know, is just, is it something I need to worry about 10 years from now? Or I just put it in the ‘who cares’ file and move on?’
Veteran P-V2	‘Well I had never heard of lung nodules to begin with. So I didn’t know what they were and I still don’t really know. I looked- I did a little research on them but I can’t really get a picture exactly of what they do or what they are.’
Veteran K-V2	‘I don’t know nothing about it, just what they tell me. I don’t have any effects from it that I know of. Yeah I’d like to know myself what’s going on with it.’
	
*Gain in knowledge*	
Veteran B-V2	‘Yeah, [the PCP] explained what calcification meant, that that was an indication of how you can get nodules, why it’s likely that what I have is from other things that occurred earlier.’
Veteran G-V2	‘I don’t know…I didn’t know what a nodule was, it took me a long time till I came in and talked to you [interviewer].’
Veteran F-V2	‘In discussing it with my primary care, [the PCP] sort of just said that that seems to have been there before but, you know, that if nothing happened in terms of growth, in terms of anything serious, that [the PCP] would say that there’s no concern about it.’

Abbreviations: PCP, primary care provider; V2, visit two.

**Table 3:** Participants’ emotional reactions to a pulmonary nodule

*Persistent distress*	
*General*	
Veteran O-V2	‘Yeah. Those [nodules] I was definitely worried about. I really was. Yeah. It was kind of, you know, off and on. I’d think about it. It would get me a little depressed.’
Veteran D-V2	‘No I kinda carried it [stress] the whole time. And things triggered it. You know seeing stuff on TV talking about cancer, just hearing things triggered it.’
*Length of follow-up*	
Veteran Q-V2	‘Because from the first initial CT exam to the last one there was a lot of time there that… you be thinking about this and you just wanna hurry up and know what’s happening with your body.’
Veteran L-V2	‘The other part of this is, I thought like, ‘well I’m waiting a year,’ and anything that I found out if there is an issue, that the sooner you deal with it the better off you are, and I thought waiting a year for another x-ray or scan is like way too long.’
	
*Decreased distress over time*	
*General*	
Veteran L-V2	‘Well I would say the first month or two was probably 6 or 7 [distress level], and then it dropped down to probably 3 or 4, and then it went down probably recently more like a- when I saw my primary care physician again I’d say it was probably just curiosity, it was probably more like 1 or 2.’
Veteran D-V3	‘Well no, you know I worried about it a little bit but no, I wasn’t freaked out like I was the first time [interview].’
*Lack of symptoms*	
Veteran F-V3	‘No because it’s something that doesn’t bother you. You know? When something bothers you of course then you know.’
*Favourable result*	
Veteran I-V2	‘Well the relief was that they said it was better, that the results looked better than before.’
*Cues from PCP*	
Veteran H-V3	‘Um, [the PCP] was very matter of fact about it. Uh, [the PCP] said they didn’t think it was [cancer], [the PCP] didn’t think it was, but we’d keep an eye on it. And I wasn’t feeling, you know, bad or anything and I said, ‘ok.’’

Abbreviations: CT, computed tomography; PCP, primary care provider; V2, visit two; V3, visit three.

**Table 4:** Patients’ suggestions for improvement in nodule discussions

*Mode of communication*	
Veteran F-V2	‘Well of course somebody directly speaking to you about it would be the best way. You know, I think that sending you some sort of a letter about it, I don’t think that would be the way to do it because the letter could get lost, something like that, so I think that you need to have a verbal discussion.’
Veteran P-V2	‘It would have been nice for somebody to sit down with me and explain to me exactly what a nodule is, what it does, and so forth.’
	
*More information*	
Veteran Q-V2	‘No, I don’t feel I have enough information…If there was a little pamphlet or something that would explain nodules or how it’s caused or what it can do or what it can lead to… I mean if I would have gotten a pamphlet like that … then that would have been very helpful to me.’
Veteran B-V3	‘I want to know everything; I don’t care if it’s upsetting. If I have reason to be upset, then let me be upset. Let me decide how upset I want to be. But don’t not tell me information. Because not knowing scares the crap out of me.’
	
*Cancer risk*	
Veteran C-V2	‘Put down odds or whatever [for cancer]. And why you take a year or so in between CT scans, and the reason behind it and stuff like that.’
Veteran D-V3	‘So those were my first thoughts [cancer]. If you could do that [report cancer risk] in a letter that said, ‘you have a 2% chance,’ or, ‘this is really rare,’ somehow downplay it, minimalize it, whatever.’
	
*Future Plan*	
Veteran F-V3	‘And of course if it’s something that they tell me, ‘Well things haven’t changed from last year,’ that’s still sort of an ominous thing. Because what happens if it does change? How do we address that specific problem in the future if it does change?’

Abbreviations: CT, computed tomography; V2, visit two; V3, visit three.

**Table 5:** Communication strategies to improve patient-centred care

*What patients want to know*	*Clinician communication strategies*	*Examples*
*Information*		
‘What is a nodule’	Provide relevant nodule information that relates to risk prediction (e.g., lack of growth decreases malignancy risk) Review chest imaging Provide written or online information	‘You have a ‘spot’ in the upper part of your right lung- let’s review the chest CT so you see it for yourself’
‘What is my lung cancer risk’	Explain personalised lung cancer risk using decisional aids, data depicted in pictures or summary tables Evaluate patients’ understanding of the concepts presented	‘Because you smoked for 50 years, there is a 5% chance this nodule is cancer. In other words out of 100 people—5 would have cancer.’ Provide link to a nodule risk calculator (e.g., http://reference.medscape.com/calculator/solitary-pulmonary-nodule-risk)
‘What are the next steps/future plan’	Describe the follow-up plan in detail including possible steps if things change (e.g., biopsy for growing nodule) Outline key imaging dates and subsequent office visits or telephone calls and provide a copy Smoking cessation guidance if applicable, framed as a ‘teachable moment’	‘I have ordered a repeat CT scan in 1 year. We will contact you a few days after the CT so we can discuss the results and next steps in management.’ ‘It’s great that your nodule is very unlikely to be lung cancer but now is a good time to quit smoking so your chances of getting cancer in the future will be even lower.’ Provide link/referral to smoking cessation interventions
		
*Emotional response*		
‘How worried should I be’	Elicit emotional responses to the information presented Provide reassurance and resources to decrease distress Enable the patient with persistent concerns ways to easily contact a clinician	‘What worries you most about this nodule?’ ‘It’s normal to be very distressed when there is even a small possibility of lung cancer.’
		
*Shared decision-making*		
‘What are my options’	Explain rationale for active surveillance Explain that other options are available but not recommended because the harms usually outweigh the benefits Address that patients often value knowing whether the nodule is cancer and prefer a more immediate answer, however, biopsies and functional imaging seldom provide this answer for small nodules Enable the patient to participate in the decision	‘This nodule is so small and the chance for cancer is so low that the best way to find out what it is- is to wait and get another CT scan. Waiting is the best option right and will not limit your treatment options later.’ ‘We can talk about biopsies and other procedures but in general, they hurt many more people than they help.’ ‘How are you feeling about waiting 6 months for your next CT scan?’

Abbreviation: CT, computed tomography.
