# Supplementary material for: ‘I still don’t know diddly’: a longitudinal qualitative study of patients’ knowledge and distress while undergoing evaluation of incidental pulmonary nodules
Source: NPJ Prim Care Respir Med. 2015 Apr 16;25:15028–. doi: 10.1038/npjpcrm.2015.28 (PMC4532158; doi:10.1038/npjpcrm.2015.28)
Supplement: Supplementary Tables [file npjpcrm201528-s1.doc]

Supplemental File: Patient Follow-up Interview Guide

**Follow-up Interview Questions**

1. What is your overall opinion of the medical care at the VA?
2. On ____________________ you underwent a follow-up CT scan of the chest to examine the lung nodule that was discovered on __________________.
3. Tell me about how you were last notified about the result of your followup chest CT.
   1. How were you told?
   2. What information were you given?
   3. Who talked to you about this?
   4. What were your biggest concerns about this information (If patient does not understand that this nodule could be cancer, terminate interview.)
4. What is your understanding of what the long-term plan is for getting more information about this nodule? How has your understanding changed about the followup plan?
5. Which clinician has talked to you most about the plan to evaluate your lung nodule?
6. Do you feel like you have enough information about this plan?
7. What makes you most uncomfortable about this plan?
   1. What has caused you the most worry?
   2. What are the most reassuring things you were told?
   3. Were there particularly ways in which the doctors and nurses talked to you that increased your distress?
   4. How about ways that decreased your distress?
8. Were you satisfied with your role in deciding what to do about your pulmonary nodule?
9. The process for evaluating this nodule may take up to two years.
   1. What has helped you most in coping with this process and uncertainty?
10. Who among the hospital personnel supported you most during this process?
    1. What did they say that was most supportive?
11. Looking back, how would you have wanted the doctors and nurses to talk to you differently about this lung nodule?
12. Does having this nodule change any other decisions you have made in your life?
    1. Has it affected your overall quality of life?
13. At any point since this lung nodule was discovered, did your clinician discuss stopping smoking?
    1. If yes, what did your doctor or nurse say?
    2. Does having the lung nodule affect your interest in quitting smoking?
    3. If you are less interested, why?
    4. If more interested, why?

14. We have found that some patients would like more information about their nodule. Let’s talk about some specific information you think might be helpful for other patients with nodules.

15. We have found that some patients either were not informed about their nodule or can’t remember being informed. How would you feel if that happened to you? How should the research team contact patients who may not have been first contacted by their doctor?

16. What else would you like to say about your feelings or the care you receive for your lung nodule?

**Stop time ____ ____**

**Duration**  ____ ____ (minutes
